# Haematopoietic stem cell transplantation for children and young people: is there a role for prehabilitation? A scoping review

**DOI:** 10.1007/s00520-025-09988-4

**Published:** 2025-11-29

**Authors:** Debbi Rowley, Raquel Revuelta Iniesta, Katharine Patrick, Sarah Massey, Peter Wright

**Affiliations:** 1 Haematology and Oncology, Sheffield Children’s Foundation Trust, Clarkson Street, Broomhall, Sheffield, S10 2TH UK; 2 Children’s Health and Exercise Centre, Faculty of Public Health and Sports Sciences, Medical School, University of Exeter, Exeter, UK; 3 Illingworth Library, Sheffield Children’s Foundation Trust, Sheffield, UK; 4 Oxford Brookes University, Oxford, UK

**Keywords:** Pediatrics, Haematopoietic stem cell transplantation, Childhood cancer, Physical functioning, Nutrition

## Abstract

**Purpose:**

Prehabilitation interventions show positive outcomes for adults undergoing haematopoietic stem cell transplantation (HSCT). Their impact on paediatric populations is less clear. This scoping review describes current prehabilitation literature, intervention feasibility, and effectiveness in improving health outcomes for children following HSCT.

**Methods:**

Following the Joanna Briggs Institute methodology, a comprehensive search of databases (AHMED, CINAHL, Cochrane library, EMCARE, MEDLINE, PsycINFO, PubMed) and grey literature was conducted. Key search terms included “HSCT”, “prehabilitation” (physical functioning, nutritional status, health behaviours), “children/young people” and “health outcomes”. Data related to review aims was extracted.

**Results:**

From 134 studies, nine met inclusion criteria (three (33%) on physical functioning, six (67%) examined nutritional status, 0 health behaviours). Eight (89%) were retrospective, with one prospective providing baseline data from a RCT.

Pre-HSCT muscle strength, endurance, balance, and quality of life are lower than age-matched peers. Higher pre-HSCT muscle strength (hip flexion *p* = 0.002) and physical function (10 m walk *p* = 0.004, time-to-rise-from-floor *p* < 0.001) correlate with improved post-HSCT recovery. Underweight (*p*-values 0.035–0.044) and overweight status (*p*-values 0.0021–0.0413) are associated with adverse clinical outcomes. Feasibility of assessment is demonstrated with no adverse events recorded.

**Conclusion:**

Initial results are promising, with a clear need for pre-HSCT assessment in clinical settings; however, no conclusions on the effectiveness of prehabilitation can be drawn. Findings emphasise the need for further research, supporting grant applications for intervention protocols.

**Graphical abstract:**

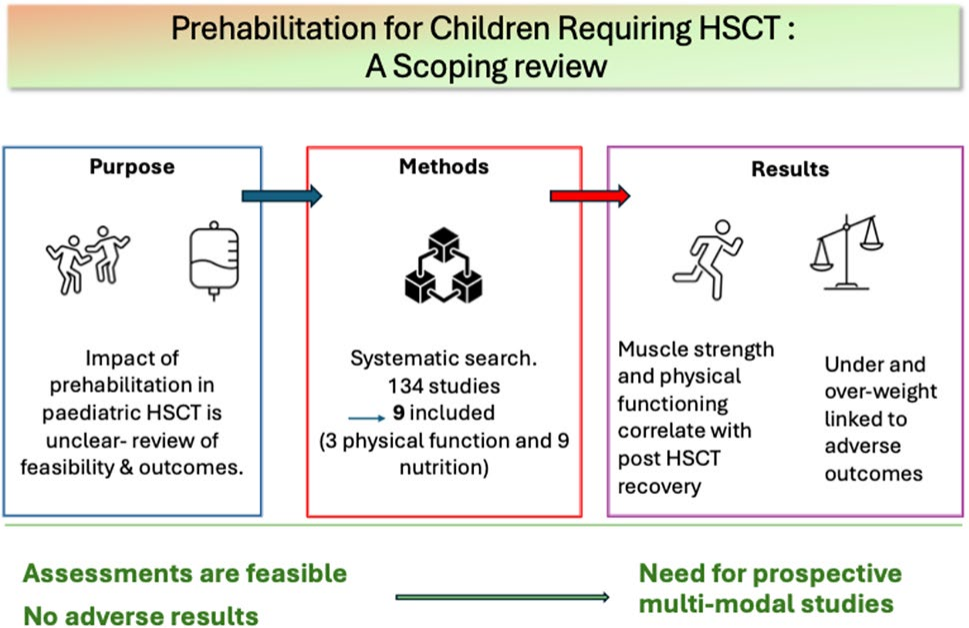

**Supplementary Information:**

The online version contains supplementary material available at 10.1007/s00520-025-09988-4.

## Introduction

The European Society of Blood and Bone Marrow Transplantation reported 5368 children received haematopoietic stem cell transplantation (HSCT), in 2019, across 241 centres in Europe [1]. HSCT is typically used in children and young people (C&YP) that have high risk malignancies where there is high potential of relapse following standard chemotherapy regimens, [2]. Other indications include benign haematology, bone marrow failure and immunodeficiencies [3]. Prognosis for C&YP undergoing HSCT for cancer has been associated primarily to cancer type, severity/grading and type of transplant. Age at transplant, transplant type and total body irradiation have been associated with long-term side effects for those requiring HSCT for a non-malignant condition, [4]. Modifiable prognostic factors for event free survival are still unclear as existing literature is scarce, largely retrospective and heterogenous [5–7].

C&YP face significant challenges in terms of physical, nutritional, and psychological wellbeing, both prior to, during, and post HSCT caused by both short- (e.g., acute infections, veno-occlusive disease, Transplant-Associated Thrombotic Microangiopathy, and acute graft versus host disease) and long-term complications (e.g.; chronic graft versus host disease, ischaemic heart disease, poor nutritional status, and low bone mineral density) [8]. Survivors of childhood HSCT carry an increased risk of endocrinopathies, metabolic syndrome, musculoskeletal disorders, cardiopulmonary disease, neurocognitive effects, chronic graft versus host disease, and further malignancies [9–11]. Poor nutritional status (malnutrition or obesity based on BMI Z-score and body composition [12]), frailty, and psychological issues can affect children’s health, recovery, and quality of life [13], though their impact after HSCT is less clear.

Prehabilitation is a proactive multi-modal intervention that involves preparing for upcoming medical interventions to enhance physical and mental readiness, improve outcomes, and accelerate recovery [14]. Prehabilitation programmes are variable and not well established in clinical practice, but gold standard approaches involve a multi-disciplinary/holistic intervention tailored to the individual’s needs. Commonly, they include exercise and/or physical activity programmes with or without nutritional advice and other healthy lifestyle advice, for example, smoking cessation [15]. Most oncology prehabilitation research has investigated the effects on the prevention of complications prior to surgery in adults [16].

Research in adult HSCT populations has demonstrated that those who are more physically active prior to transplantation have better outcomes. The concept of implementing prehabilitation for adults undergoing HSCT has gained traction in the literature, with publications on the topic emerging since 2019 [17, 18]. These studies are generally uni-modal in delivery, with an emphasis on physical activity and exercise. For example, a retrospective review of 207 adult patients demonstrated that higher performers experienced significant reduction in pain, fatigue, and distress post HSCT, and when controlled for transplant type, a significant reduction in mortality [19]. Nutrition, as a prehabilitation intervention for HSCT, has largely been neglected. Literature searches [14]and retrospective studies [20]have highlighted the negative impact HSCT has on nutritional status and the lack of standardisation of any interventions. Early assessment is recommended to enable prevention and treatment, supporting the need for inclusion of nutritional prehabilitation [18, 20].

Evidence supporting physical activity during and post-HSCT in C&YP has demonstrated safety and beneficial outcomes [21]. No scoping or systematic review of prehabilitation in the C&YP population, including physical activity, exercise, nutrition, and other health behaviour interventions has been registered or published. A narrative review by Sandblom et al. [23] uncovered no original studies about physical activity prehabilitation. Narrative reviews are not systematically rigorous; thus, this scoping review aims to build on that review by systematically mapping the breadth of the literature (including grey literature) for C&YP who require HSCT as part of their treatment. Additionally, it will summarise the feasibility and effectiveness of interventions that produce positive outcomes. It is intended that findings will contribute to the design and implementation of evidence-based clinical pathways.

## Methods

Due to the anticipated small volume of evidence, a scoping review was identified as the most appropriate methodology for this review. Conducted in accordance with the Joanna Briggs Institute (JBI) methodology for scoping reviews [23]and the PRISMA-SCR extension guideline [24], it enabled navigation across multi-disciplinary themes whilst identifying gaps in the literature. The research question and eligibility criteria have been defined using the Population-Concept-Context (PCC) framework [25] and are presented in Table1.

**Table 1:** Inclusion and exclusion criteria

Review question: Question: Does prehabilitation improve health outcomes for children requiring haematopoietic stem cell transplantation following a diagnosis of cancer?		
	Inclusion	Exclusion
Participants	Children aged up to 18 years with cancer requiring treatment with HSCT	Children requiring HSCT for any other reason
Context	Global literature Any language—however, the abstract must be available in English with sufficient information to understand the methodology and results	
Concept	Prehabilitation Including physical activity/exercise, nutrition, health behaviour change interventions prior to HSCT	Rehabilitation (during or post HSCT)
Sources of evidence	Quantitative studies (Including both randomised and non-randomised control trials) Qualitative studies Mixed methods studies Observational studies Reviews/case series Individual case reports Conference abstracts Text and opinion papers Grey literature	
Time frame	1980 until October 3rd 2024	Prior to 1980, due to the establishment of the bone marrow donors worldwide programme

A comprehensive search strategy and protocol was developed and registered on Figshare, reference number: 26720929.v1 [26] prior to the commencement of the search in September 2024. Search terms were developed in conjunction with topic experts and reviewed by each member of the authorship. Key search terms included “HSCT”, “prehabilitation” (physical functioning, nutritional status, and health behaviours), “children/young people” and “health outcomes”. A full expanded list of the key terms is found in the protocol. All identified search terms were adapted for the included sources. The search applied Boolean operators (‘AND,’ ‘OR,’ and ‘NOT’) and applicable truncations (*). Search terms were not revised following stage one of the search as no further terms were identified. Please see Appendix 1 as Electronic Supplementary Material for an example of the search strategy used in Embase. Full search strings used for all identified sources can be requested from the corresponding author.

All identified citations were collated and uploaded in Zotero [27], with duplicates removed. Abstracts were screened by two reviewers (DR and RRI) according to the eligibility criteria. Sources identified as relevant were retrieved in full and further assessed against the inclusion/exclusion criteria by two reviewers (DR and RRI). Any disagreement between reviewers on study inclusion was discussed with a third reviewer (PW) for decision. Primary literature was pulled from reviews to eliminate the possibility of double counting evidence.

Articles with an adult population that included young people were reviewed. Of the three articles that included young people, two did not recruit any young people under the age of 18. One paper included one young person aged 17 years, but this data was not extractable from the rest of the data, and therefore it was decided not to include the article.

Article that included C&YP requiring HSCT for either cancer or non-malignant conditions were included in the study if there was demographic data stating that there were C&YPs with a cancer diagnosis included in the study.

Evidence extraction followed a two-phase process into pre-established proformas. Phase 1 included the extraction of the descriptive/bibliographic information, e.g., author, year of publication, country of origin, aims, population, sample size, and methods. Phase 2 involved the extraction of findings that align with the aim and objectives of this review. An evidence extraction form was developed and reviewed by all authors and can be provided on request. Data was extracted by DR and reviewed by RRI to ensure consistency. Extracted evidence was themed to each research question and summarised descriptively.

## Results

**Fig. 1:**
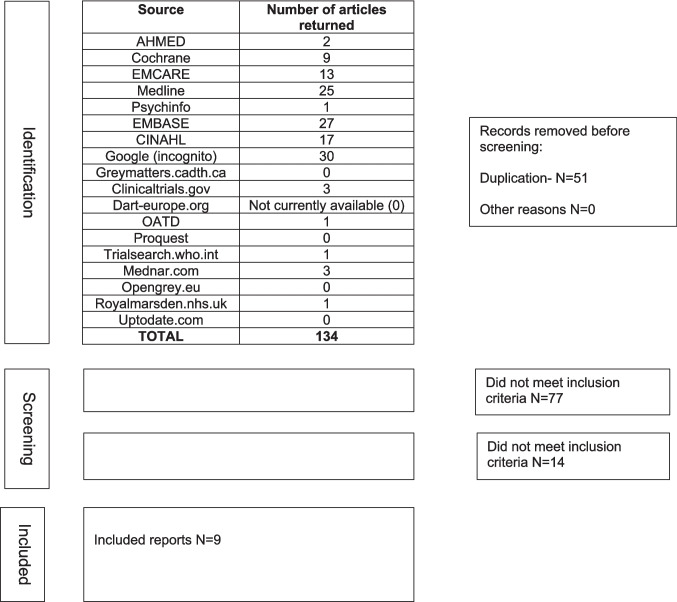
PRISMA diagram

Our search identified 134 articles plus an additional 17 from reference lists. Of these, 51 were duplicate articles, and therefore 100 were screened against inclusion criteria. Nine articles (9%) met the inclusion criteria, with articles being excluded primarily due to the article reporting physical activity, nutrition, or health behaviour interventions during or post HSCT or being related to the adult population. Results of the scoping review can be seen in the PRISMA diagram, Fig. 1. Of the included articles, three (33%) related to physical functioning and are presented in Table 2, and six (67%) delivered nutritional results and are presented in Table 3. No studies on other health behaviours have been identified. No studies combined physical functioning, nutrition, and behaviour/psychological interventions. All studies included children requiring HSCT for both malignant and non-malignant conditions.

**Table 2:** Studies investigating physical activity or exercise and clinical outcomes

Author, year, country, (ref)	Aims	Study design	Population	Exposure	Outcomes	Measures	Key results
den Hartog E. et al. (2024) Netherlands [28]	Is pre-HSCT physical performance associated with post-HSCT physical performance?	Single centre retrospective cohort analysis	*N* = 77 Age range 3–18yrs Females 36% Allogeneic HSCT	Standard of care physiotherapy assessment pre-HSCT and in-patient physiotherapy assessment at around 100 days post HSCT	Muscle strength	Handheld dynamometry (MicroFET2, Procare), eccentric break-technique protocol in standardised positions Isometric strength: hip flexion, knee extension, and ankle dorsal-flexion (average of both legs)	Hip flexion strength pre-HSCT positively associated with post-HSCT regression coefficient 0.59 (*p* = 0.002) Hip flexion mean and SD: pre-HSCT 160.4 (70.2), post-HSCT 140.1(54.1) Knee extension mean and SD: pre-HSCT 199.4 (78), post-HSCT 195.8 (70.2) Ankle dorsal-flexion mean and SD: pre-HSCT 162.1 (61.2), post-HSCT 159.3 (52.0)
						Handgrip- sitting with elbow at 90° using Jamma HHD (Sammons)- in kg	Dominant mean (SD): pre-HSCT 25.2 (17.8), post-BMT 19.0 (8.6) Non dominant mean (SD): pre-HSCT 23.8 (15.5), post-HSCT 17.0 (7.6)
					Muscle mass	bio-impedance. appendicular skeletal muscle mass (ASMM) using Tantia MC-780	ASMM pre-HSCT positively associated with post-HSCT, regression coefficient 0.89 (p < 0.001)
					Physical functioning	Time to rise from floor (TRF) from cross-legged position, 10 m walk (10-MWT) time to walk 10 m at a comfortable pace, and timed up and downstairs (TUDS) time to ascend and descend 10 steps	-TRF pre-HSCT positively associated with post-HSCT, regression coefficient 1.21 (p < 0.001) −10-MWT pre-HSCT positively associated with post HSCT, regression coefficient 0.62 (*p* = 0.004) -TUDS pre-HSCT positively associated with post HSCT, regression coefficient 0.89 (p < 0.001)
Lenker, H. and Foley M. (2019) USA [2]	Overview and discussion of a physical therapy-led pre-transplant evaluation	Single centre prospective baseline data gathering discussion overview	*N* = 54 Age range – children aged 3 + Mixed allogeneic and autologous HSCT	Standardised pre-HSCT physiotherapy assessments		QoL. Range of motion. Muscle strength (Bruininks-Oseretsky test of motor proficiency (BOTMP-2)- strength subset), Aerobic endurance (6-min walk test). Balance (BOTMP-2- balance subset). Gait: subjectively and/or Protokinetics Zeno walkway gait analysis mat). Fatigue (Patient reported)	No adverse events Suggesting a Pre-Transplant Physical Therapy assessment would allow the identification of functional impairment, restore function, enhance QoL, and prevent secondary comorbidities
Beller R. et al. (2024) Germany [29]	Evaluate pre-HSCT fitness status against that of healthy peers (published reference values- normative data)	Single centre baseline data from an RCT- prospective data gathering	*N* = 22 Age range 4–18yrs Females 23% Allogeneic HSCT	Participants can come from differing referring centres with different pre-treatment protocols, experiencing different physiotherapy/exercise interventions prior to assessment Assessments took place 1 to 2 days prior to starting conditioning as an inpatient	Muscle strength	Hand grip strength (isometric–Jamar handheld dynamometry and MOON test) Leg extension strength (isometric maximum extension with digital force gauge-Sauter)	Hand grip strength: participants 46% lower on right, 48% lower on left than ref. values (*p* < 0.001) Leg extension: participants 45% on right (*p* = 0.002), 50% lower on left (*p* = 0.001)
					Balance	1-min static stance/whole-body posture on a wooden bar, MOON test	Participants 106% lower than ref. values (p < 0.001)
					Endurance	5 × sit-to-stand and MOON Test Cycle ergometer test (stationary bike ergometer and steep ramp test)	Sit-to-stand participants are 34% lower than reference values (*p* = 0.019) Cycle ergometer participants are 44% lower than ref. values (p < 0.001)
					Quality of life	Social, Emotional, Physical, and School Functioning (PedsQL SF15) Multidimensional PedsQL Fatigue Scale (self and parent report)	PedsQL SF15 participants scored 80% less than ref. values (*p* < 0.001) PedsQL fatigue participants scored 86% less (*p* = 0.016). Higher fatigue correlates with > inpatient days, *r* = 0.47 (*p* = 0.034)
					Number of supervised individualised exercise prior to HSCT		Range 1–52. Negative correlation between supervised exercise sessions and hand grip strength, and less deviation from ref. values (*r* = 0.43, *p* = 0.047) Number of exercise sessions predicts higher fatigue (t = −2.407, *p* < 0.05), and lower HRQoL (t = −3.763, *p* < 0.01), also predicted by inpatient days (t = −2.251, *p* < 0.05)

**Table 3:** Studies investigating nutrition and clinical outcomes in C&YP HSCT

Author, year, country, (ref)	Aims	Study design	Participants	Exposure	Outcomes	Measures	Results
Kerby E. et al. (2017) USA [30]	Does pre-HSCT malnutrition influence the development of graft versus host disease (GVHD) and mortality?	Single centre retrospective cohort analysis	*N* = 134 Age range 0–20yrs Females 56% Allogeneic HSCT	Chart review at baseline, 30, 60, and 90 days following HSCT	Nutritional markers	Body Mass Index (BMI)- 2 versions of categorisation- 1.- < 25th percentile, 25th-85th and > 85th 2.—< 5th, 5-95th, and > 95th Serum albumin (moderate malnutrition < 2.8 g/dl) and vitamin D (low < 25 ng/ml)	Low BMI alone and BMI, serum albumin, and weight loss from baseline are associated with a 3–4 × increased risk of developing severe GVHD in the next 30 days Low vitamin D is not associated with severe GVHD BMI < 25th percentile was not significantly associated with day 100 mortality (*p* = 0.19). Those with low BMI, low serum albumin, and weight loss had an increased risk of death (7% v 1%, *p* = 0.035)
					GVHD	grade I, II, III,IV (Keystone consensus grading scale- III and IV = severe)	
Kranjcec, I. et al. (2020) Croatia [31]	The impact of nutritional status on adverse events and outcomes in paediatrics following autologous HSCT	Single centre retrospective cohort study	*N* = 77 Age range 0–19yrs Females 45.5% Autologous HSCT	Chart review at diagnosis, procedure, discharge, and 3 years after the procedure	Nutritional Markers	BMI (< 5th,5th- < 85th, 85th- < 95th and > 95th percentiles). Serum albumin (> 30 g/l, 30-20 g/l and < 20 g/l)	No differences in the frequency of adverse events in the different BMI and serum albumin groups
					Adverse events	Fever, gastrointestinal toxicity, electrolyte disorders, dysglcemia (categories 1–4 as National Health Institute, National Cancer Institute and Common terminology for adverse events)	Underweight group – more prevalent hypokalemia (*p* = 0.041) and hypomagnesemia (*p* = 0.044) Children with higher BMI – less prone to severe mucositis (*p* = 0.016) and profound hypophosphatemia (*p* = 0.038)
					Time to engraftment, relapse, and mortality	Time to engraftment – neutrophil count > 0.5 × 10^9^/L for > 3 days, platelet count > 20 × 10^9^/L for > 7 days	Relapse rate and mortality did not differ significantly between BMI, although those that were underweight had a better outcome (log-rank test *p* = 0.132)
Author, year, Country, (ref)	Aims	Study design	Participants	Exposure	Outcomes	Measures	Results
Murphy, J. et al. (2024) USA [32]	Determine the feasibility and impact of the enteral nutritional programme on: Nutritional status, transplant-associated complications, and patient outcomes	Quality improvement project; pre- and post-intervention design Intervention: pre-HSCT nutritional analysis and NG or gastrostomy advised for the malnourished group	*N* = 12 Age range 1–15 years Females 58% Mixed allogeneic and autologous	Registered dietician review pre-BMT; nutritional screening and education. Those at high nutritional risk referred for gastrostomy. Or NG tubes placed typically between day −1 to + 4	Nutritional status	Weight/length z score, weight loss and mid-bicep circumference, malnutrition (Academy/ASPEN tool)	Weight change—no sig difference between groups (*p* = 0.394) Malnutrition—no significant difference between groups (*p* = 0.545)
					Transplant complication	Infection rates and GVHD (MAGIC criteria)	Infection rate—no sig difference (*p* = 0.368) GVHD- no sig difference (*p* = 1.0) in rate or severity (*p* = 0.667)
					Patient outcomes	Time to platelet and neutrophil engraftment (definitions; Centre for International Blood and Marrow Transplant Research), length of hospital stay	Length of hospital stay- no sig difference between groups (*p* = 1.0) Platelet engraftment—no sig difference between groups (*p* = 0.589) Neutrophil engraftment- no sig difference in groups (*p* = 0.818)
					Feasibility	Clinician reported satisfaction survey	Questionnaire return 45%. 88.9% agreed/strongly agreed with the project
Stetter et al. (2023) Germany [33]	Investigate the impact of BMI on clinical outcome up to 200 days post HSCT	Single centre retrospective data Annual meeting abstract	*N* = 365 Age range 0–23yrs Female 56% Mixed autologous and allogeneic	Chart data at day of inpatient admission, day + 30, and day + 200 post HSCT	Adjusted BMI percentiles		27% allo and 44% auto ascend ^3^ 1 BMI percentiles from day 0–200 46% allo and 22% auto descend ^3^ 1 BMI percentiles from day 0–200
					Incidence of side effects:	GvHD, veno-occlusive disease (VOD), mucositis, infection	BMI > 75th-97th percentile Grade 3–4 intestinal GvHD (*p* = 0.0028), VOD (*p* = 0.0413), BKV cystitis (*p* = 0.0021), viremia (*p* = 0.0081) increased frequency
					Survival		32% overall mortality. Allogeneic- *N* = 35. 34% were in BMI > 75–97 percentile. Autologous- *N* = 23. 26% were BMI < 3 percentile
White, M. et al. (2012) Australia [34]	Does pre-HSCT body weight influence overall survival?	Single centre retrospective audit (Cohort)	*N* = 113 Age range 3–15yrs Females 33% Mixed allogeneic/autologous HSCT	Chart review 9 days pre-HSCT and then for at least 3 years post-HSCT or until death	Ideal body weight % Overall survival	Growth charts for children by Centres for Disease Control and Prevention. Underweight < 90, Ideal weight 90–110, overweight > 110 (cutoffs from children’s oncology group nutrition committee)	13% underweight, 41% overweight prior to HSCT Overweight sig. less likely to survive than ideal weight (hazard ratio 1.91, 95% confidence interval, 1.10–3.31) No sig increase in survival for underweight patients compared with ideal weight, (hazard ratio 1.47, 95% confidence interval, 0.57–3.79)
Author, year, Country, (ref)	Aims	Study design	Population	Exposure	Outcomes	Measures	Key Results
Tavil, B. et al. (2012) Turkey [35]	Investigate the effects of pretransplant nutritional status, habits and content on clinical outcomes	Single centre prospective cohort study Length of time (NR) Methods: Dietary assessment Self-report Semi-quantitative food frequency questionnaire of 47 food items 24-h dietary recall (tool NR) (tool NR) The Nutritional Information System (BeBis) to assess energy and nutrients content	*N* = 41 Age range 1–17 years F (31.7%) M (68.3%) Allogeneic	HSCT Neutropenic Diet (throughout transplant until neutrophil engraftment) Education on food choices throughout hospitalisation (reference NR)	Nutritional status	BMI–categorised pre-transplant (WHO 2007 percentiles) Characteristics at baseline: Underweight (< 5th C) = 12.2% Risk of underweight (5—< 15th C) = 12.2% Healthy weight (15th < 85th) = 53.6% Overweight (85th < 95th) = 9.8% Obese (≥ 95th) = 12.2% Mean TEI and nutrient intakes before HSCT compared with Dietary Reference Intake (%) Probiotics and prebiotics food intake before HSCT (g/day)	Associations of BMI with clinical outcomes (X^2 –^test): Frequency of complications- 60% for risk of underweight or already underweight, 41% for healthy weight, and 67% for overweight or obese (P > 0.05) Duration of febrile neutropenia mean ± SD, 4.9 ± 4.6 days for risk of underweight and underweight, 3.2 ± 2.8 days for healthy weight, and 3.3 ± 3.1 days for overweight or obese (p > 0.05) Day of neutrophil engraftment—mean ± SD, 15.4 ± 4.1 days for risk of underweight and underweight, 15.9 ± 3.2 days for healthy weight, and 14.9 ± 2.5 days for overweight or obese; (*p* > 0.05) Day of platelet engraftment mean ± SD, 22.6 ± 8.7 days for risk of underweight and underweight, 23.2 ± 7.2 days for healthy weight, and 21.6 ± 6.4 days for overweight or obese (*p* > 0.05) Correlation between dietary intake (DRI%) (baseline data/during study period unclear) and clinical outcomes Correlation between some nutritional factors and neutrophil engraftment day Amount of consumed soluble fibre *r* = –0.393; *p* = 0.01 Amount of consumed iron – *r* = 0.365; *p* = 0.02 Duration of breastfeeding – *r* = 0.388; *p* = 0.03 Amount of consumed fermented bread *r* = –0.506; *p* = 0.02 Amount of consumed bulgur *r* = –0.477; *p* = 0.003 Correlation between some nutritional factors and duration of febrile neutropenia: Amount of consumed yogurt *r* = –0.422; *p* = 0.009 Amount of consumed onion intake *r* = –0.457; *p* = 0.008 Amount of consumed fruited cream cheese, *r* = 0.491; *p* = 0.04 Amount of consumed haricot bean *r* = 0.338; *p* = 0.04 Correlation between some nutritional factors and duration of TPN intake: Amount of consumed onion intake *r* = –0.659; *p* = 0.04 Amount of consumed parsley *r* = –0.733; *p* = 0.03
					Clinical Outcomes	Severity of oral mucositis (common toxicity criteria) Presence and severity of acute GvHD (Glucksberg criteria) Infections- positive cultures, abnormal diagnostic results including radiological findings, Fever, Neutropenia Neutrophil Engraftment Death/Alive without disease	Nutritional status: no effect on complication frequency (p > 0.5), duration of febrile neutropenia (p > 0.5), day of neutrophil or platelet engraftment (p > 0.5) Negative correlation: day of neutrophil engraftment and consumed soluble iron (*r* = − 0.506, *p* = 0.2) and fibre (*r* = − 0.393, *p* = 0.012) Nutrients associated with shorter time to engraftment: breastmilk (*r* = − 0.388, *p* = 0.03), bazlama (*r* = − 0.506, *p* = 0.02), and bulgar (*r* = − 0.477, *p* = 0.003) Febrile neutropenia – shorter durations associated with yoghurt (*r* = − 0.422, *p* = 0.009) and onion (*r* = − 0.457, *p* = 0.008), sufficient vit C (*p* = 0.03). Longer duration readymade fruited cream cheese (*r* = 0.491, *p* = 0.04) and haricot bean (*r* = 0.338, *p* = 0.04) Lower parsley consumption in those with higher complications (*p* = 0.02)

### Physical functioning

In comparison to healthy peers, C&YP requiring HSCT exhibited significantly lower muscle strength (right leg extension mean for the test group 149.6 (newtons) compared with healthy peers 229.2 (newtons) and hand grip strength in kg, mean 11.9 in the test group compared with 19.3 in healthy peers), endurance (sit to stand mean 8.4 repetitions in the test group compared with 6.5 in healthy peers), and static balance (ground contacts 16.6 in the test group and 8.9 in healthy peers), with handgrip and leg extension strength up to 50% below reference values. Fatigue (mean PEDSQL score 72.1 compared with 84.2 in healthy peers) and poorer quality of life scores (PEDSQL SF15 mean score of 68.6 compared with 83.1 in healthy peers) were also evident and correlated with longer inpatient stays (fatigue: *r* = 0.47). A greater number of supervised pre-HSCT exercise sessions was correlated with improved handgrip strength (*r* = 0.43, *p*= 0.047) [29].

Pre-HSCT physical performance was consistently associated with post-HSCT outcomes. Greater pre-HSCT hip flexion strength (regression coefficient = 0.59, *p* = 0.002) and muscle mass (regression coefficient = 0.89, *p* < 0.001) correlated with better post-HSCT performance. Hip flexion, knee extension, ankle dorsal-flexion, and hand grip strength all reduced from pre-HSCT to post-HSCT assessment; however, statistical significance was not reported. Functional measures such as time to rise from the floor (regression coefficient = 1.21, *p* < 0.001), 10-m walk time (regression coefficient = 0.62, *p* = 0.004), and stair climbing ability (regression coefficient = 0.89, *p*< 0.001) were also predictive of post-HSCT performance [28].

A physical therapy-led pre-HSCT evaluation was suggested as a valuable tool for identifying functional impairments and guiding interventions [2]. No adverse events were identified in the included articles. None, however, explored the feasibility of delivering a pre-HSCT physical functioning intervention.

### Nutrition

Pre-HSCT nutritional status was identified as a potential determinant of post-HSCT complications, with BMI reported in 83% of the nutrition studies. Nutritional deficiencies—specifically low BMI, hypoalbuminemia, and malnutrition—were associated with an increased risk of adverse outcomes, including severe graft-versus-host disease (GVHD) [30]. Tavil et al. [35]reported that 60% of C&YP at risk of, or already underweight, experienced complications, compared to 41% in the healthy weight group. In contrast, Kranjcec et al. [31] found no significant differences in complication rates across BMI categories. Underweight C&YP demonstrated higher rates of electrolyte imbalance (*p*= 0.041–0.044) [31]. Variability in outcome measures and BMI classification across studies limited data synthesis for this subgroup.

Elevated BMI was associated with both protective and adverse outcomes. Higher BMI correlated with reduced incidence of mucositis (*p* = 0.016) and metabolic complications (*p*= 0.038) [31]. Conversely, overweight C&YP showed increased rates of severe intestinal GVHD (*p* = 0.0028), veno-occlusive disease (*p* = 0.0413), and infections (*p*= 0.0021–0.0081) [33]. Tavil et al. [35] reported complication rates of 67% in overweight patients, compared with 41% among those of healthy weight.

Mortality was an outcome in 83% of the studies. White et al. [34]reported no significant difference in survival between malnourished and healthy weight C&YP (HR = 1.47), a finding corroborated by Kerby et al. [30], who found that a BMI below the 25th percentile was not significantly associated with 100-day mortality (*p*= 0.19). Kranjcec et al. [31] similarly reported no significant differences in mortality between BMI groups. However, C&YP with low BMI, hypoalbuminemia, and weight loss exhibited higher mortality rates (7% vs. 1%; *p*= 0.035). Notably, White et al. [34] found that severely overweight C&YP had nearly twice the risk of mortality compared to those of ideal weight (HR = 1.91), an association not reported in other studies.

Dietary intake prior to HSCT was also linked to recovery trajectories. Higher consumption of soluble fibre, iron, breastmilk, and traditional Turkish foods (e.g., bulgur, fermented bread) was associated with faster neutrophil engraftment, whereas diets high in processed foods (e.g., ready-made fruited cheese) and low in vitamin C were linked to prolonged febrile neutropenia [35].

Only one study evaluated a nutritional intervention. A structured enteral nutrition programme was deemed feasible; however, it did not significantly impact transplant-related outcomes such as hospital length of stay (control group median 32.5 days compared with test group 31 days), platelet engraftment (control group median day 22 compared with test group day 20.5), or neutrophil engraftment (control group day 14 compared with test group day 15.5) [32].

### Health behaviours

No studies addressing other health behaviours such as smoking cessation or sun protection behaviours were identified through the search.

### Multi-modal studies

No studies were identified that combined physical functioning, nutrition, and/or other health behaviours.

## Discussion

Few studies were identified that met inclusion criteria supporting the understanding that prehabilitation for C&YP is in its infancy. The review has described the current literature related to prehabilitation themes (nutrition, physical functioning, and health behaviours); however, no feasibility studies were found. This paucity of evidence leads to no conclusions being drawn on the effectiveness of prehabilitation interventions.

Methodological variation was evident across all publications. Physical functioning studies varied in design, including retrospective data, RCT baseline data, and descriptive assessments. Most nutritional studies used retrospective cohort designs (*n*= 41–365), offering larger samples but were limited by control over outcomes measured and lack of standardisation [30, 31, 33–35]. One small intervention study (*n*= 12) showed no significant group differences, though clinicians found it feasible and clinically meaningful [32]. All studies were single-centre, limiting generalisability and external validity and increasing bias [36]. This inconsistency in outcomes measured and methodology prevents meta-analysis. Each of the studies acknowledged these limitations and called for future research with larger, multi-centre designs.

Significant heterogeneity also existed across the studies, with participants aged 0–23, presenting with various malignant and non-malignant conditions and transplant type. Wide age ranges reflect the small population of C&YP requiring HSCT, necessitating broader inclusion criteria. Whilst this diversity supported recruitment, it has limited generalisability and interpretation. All studies emphasised the need for larger, multi-centre, prospective trials.

Outcomes measured varied but included muscle strength and functional assessments in physical functioning studies, and mortality (*n* = 3) and treatment-related toxicities (*n*= 6) in nutritional studies. Two of the three physical functioning papers [28, 29]used hand grip dynamometry and isometric leg extension, with hand dynamometry noted for its strong reliability and status as a gold standard [37]. Functional measures lacked consistency, limiting interpretation. Pre-HSCT nutritional status was most often assessed by weight and BMI (used in 87% of studies), with serum albumin (*n* = 2) and vitamin D (*n*= 1) also reported. Although all papers examined toxicities in relation to nutritional status, none assessed their impact on quality of life. Overall, the studies reflect a shared recognition of the importance of evaluating pre-HSCT physical functioning and nutritional status, aligning with literature from the adult populations [18, 20].

Despite these limitations, findings emphasise the clinical importance of early, targeted physical functioning and nutritional assessment for C&YP undergoing HSCT. Parallels across age groups and clinical settings support further development of feasibility and integration of structured prehabilitation protocols. The authors would therefore recommend the future development of feasibility studies that include physical functioning measures and nutritional status.

### Physical functioning

Although the number of published studies is limited and outcome measures vary considerably, existing evidence reports generally positive results. None of the identified papers reported adverse events whilst assessing physical functioning, and current evidence suggests that exercise interventions during and after HSCT are safe in children [21, 38]. A lack of adverse events associated with exercise and physical activity training is also evident in prehabilitation literature for adult HSCT [18].

More active lifestyles at all stages of the HSCT pathway, including prehabilitation, maintenance, or habilitation during the inpatient phase and then rehabilitation, align with public health drivers [39]. Evidence suggests that physical activity and exercise programmes during and after HSCT are impactful on short- and long-term complications and quality of life [40–42]. Taking into consideration the presented evidence alongside emerging evidence in the adult HSCT population [14, 17, 18], prehabilitation for C&YPs including physical training could be conceptualised as having a positive impact on outcomes and should therefore be further explored.

Although only one small intervention study is currently published, international guidance on exercise and physical activity for C&YPs with cancer is [43]and could therefore be utilised to develop safe programmes for delivery and measurement of feasibility and effectiveness. Clear guidance or protocol, including which measures are of highest clinical value and provide the most useful data in terms of intervention effectiveness, would support future research [44].

### Nutrition

Nutrition prehabilitation research is in its infancy and firm results are precluded by the heterogeneity of the C&YP population being admitted for HSCT. Nevertheless, low BMI alone did not appear to affect overall mortality [30, 34]; however, malnourished C&YP experienced higher complications, specifically higher GVHD severity and electrolyte imbalances [30, 31, 35]. These findings mirror outcomes seen in adult HSCT recipients, where malnutrition and sarcopenia are independently associated with poorer transplant tolerance, higher infection rates, and lower survival [45, 46]. Similar trends are evident in C&YP with cancer but not requiring HSCT and those with chronic conditions such as cystic fibrosis or inflammatory bowel disease, where nutritional status is a strong predictor of treatment response, hospital stay duration, and complication rates [47, 48].

Obesity is a preventative risk for adult cancers and cardiovascular disease and is a key public health driver internationally [49]. C&YP cancer survivors are at increased risk of obesity due to the treatment they receive and its effects on lifestyle including sedentary behaviours and dietary intake [50]. This, coupled with C&YP requiring HSCT being restricted to long periods of isolation and treatment effecting their nutritional intake, further increases the risk. Evidence suggesting that C&YPs that were obese admitted for HSCT had increased risks of severe GVHD, infections, and increased mortality rates [31, 33, 35]is matched by evidence in other C&YP cancer populations [51]. As a modifiable risk factor, obesity should therefore be addressed in prehabilitation programmes.

Pre-HSCT dietary intake may also affect recovery, with higher fibre and iron consumption linked to faster engraftment and processed foods associated with prolonged neutropenia [35]. Specificity of dietary intake recording and analysis is complex, and there continues to be a paucity of evidence; however, other studies recruiting C&YPs with cancer diagnoses have outlined the potential protective effects of some food groups, including antioxidants [52], and the importance of understanding fluctuating dietary intake has been highlighted [53].

The papers had conflicting results, with some reporting increased risk of complications or reduced survival for those in either overweight or underweight categories, whilst others reported no significant differences. Interestingly, no papers suggested that being at ideal weight would increase risk factors for complications or reduce survival. Further evaluation and feasibility of programmes and protocols to support optimal nutrition prior to C&YP HSCT is essential to understand the implications on complications and mortality.

### Multimodal

No studies were identified that concurrently addressed nutritional status and physical functioning within a multi-modal prehabilitation framework or that integrated these components with health behaviours and psychological well-being. This contrasts with evidence from adult oncology prehabilitation research [14, 17]. Nutritional strategies and physical activity can mitigate adiposity and muscle wasting, with exercise further enhancing strength and cardiorespiratory fitness. When combined, these interventions may synergistically promote lean mass accrual, reduce fat mass, and improve treatment outcomes by decreasing complications and enhancing therapy tolerance (https://www.zotero.org). The absence of multi-modal intervention development in C&YP is not unexpected; however, future research should adopt a holistic approach to assessing modifiable factors to reflect clinical realities.

## Conclusion

This review found limited research on prehabilitation for children requiring HSCT, with no randomised control trials and mostly low-quality data such as retrospective studies and expert opinions. Nutritional and physical functioning interventions were notably absent, and service user input was missing from study development. Variation in measures used prevents generalisation of results, and the statistical reporting of the data was not always optimised. Despite these limitations, the findings suggest early physical and nutritional optimisation could improve outcomes. The authors recommend further feasibility studies and intervention-focused protocols in this emerging area. These should include C&YP and their families co-designing interventions for piloting in multi-centre trials. Consideration of behavioural change evidence during intervention development is also recommended [22].

## Supplementary Information

Below is the link to the electronic supplementary material.

ESM 1 (PDF 88.9 KB)

## Data Availability

All data generated or analysed during this study are included in this published article.
