# Supplementary material for: Haematopoietic stem cell transplantation for children and young people: is there a role for prehabilitation? A scoping review
Source: Support Care Cancer. 2025 Nov 29;33(12):1149. doi: 10.1007/s00520-025-09988-4 (PMC12664845; doi:10.1007/s00520-025-09988-4)
Supplement: Supplementary file 1 — (PDF 88.9 KB) [file 520_2025_9988_MOESM1_ESM.pdf]

Appendix 1: Search strategy conducted in Embase (via OVID)

Search conducted 3rd October 2024

1 stem cell transplant.mp. (30450)
2 exp stem cell transplantation/ (204861)
3 exp hematopoietic stem cell transplantation/ (95141)
4 haematopoietic stem cell transplantation.mp. (5630)
5 umbilical cord blood transplantation.mp. (1553)
6 cord blood stem cell transplantation/ (7246)
7 bone marrow transplantation/ (57121)
8 autologous bone marrow transplantation/ (6393)
9 bone marrow cell transplantation.mp. (263)
10 bone marrow grafting transplantation.mp. (0)
11 bone marrow cancer/ (2836)
12 allogeneic stem cell transplantation/ (20124)
13 autologous stem cell transplantation/ (22356)
14 1 or 2 or 3 or 4 or 5 or 6 or 7 or 8 or 9 or 10 or 11 or 12 or 13 (276295)
15 prehabilitation.mp. (2980)
16 preoperative exercise/ (2020)
17 multimodal prehabilitation.mp. (303)
18 before.mp. [mp=title, abstract, heading word, drug trade name, original title, device manufacturer, drug manufacturer, device trade name, keyword heading word, floating subheading word, candidate term word] (2242282)
19 prior.mp. [mp=title, abstract, heading word, drug trade name, original title, device manufacturer, drug manufacturer, device trade name, keyword heading word, floating subheading word, candidate term word] (1293136)
20 rehabilitation conditioning.mp. (6)
21 aerobic exercise/ or exercise/ (387571)
22 fitness/ (45249)
23 transplantation conditioning/ (2967)
24 physical activity/ (241264)
25 transplant conditioning.mp. (1006)
26 multimodal exercise.mp. (285)
27 resistance training/ (30610)
28 strength training.mp. (9843)
29 training/ (129021)
30 mobility.mp. (289567)
31 function.mp. (4454552)
32 movement.mp. or "movement (physiology)"/ (514646)
33 rehabilitation/ or cancer rehabilitation/ or functional training/ or muscle training/ or pediatric rehabilitation/ (119668)
34 physiotherapy/ or pediatric physiotherapy/ (114239)
35 physical therapy.mp. (42603)
36 exp kinesiotherapy/ (107528)
37 exercise intervention.mp. (9292)
38 exercise program.mp. (16787)
39 structured physical activity.mp. (342)
40 preoperative exercise/ or prehabilitation exercise.mp. (2048)
41 physical exercise interventions.mp. (245)
42 nutritional support/ (24776)
43 enteric feeding/ (43149)
44 parenteral nutrition/ (36632)
45 gastrostomy/ (14637)
46 nutrition therapy.mp. or diet therapy/ (69308)
47 dietetics/ (6631)
48 dietitian/ (19507)
49 diet/ (264562)
50 food/ (82033)
51 health behavior/ (85095)
52 smoking cessation/ (72961)
53 health behavior/ or behavior change/ (136919)
54 psychological aspect/ or psychological interactions.mp. (490888)
55 behavioural counselling.mp. or counseling/

(84057)
56 15 or 16 or 17 or 18 or 19 or 20 or 21 or 22 or 23 or 24 or 25 or 26 or 27 or 28 or 29 or 30 or 31 or 32 or 33 or 34 or 35 or 36 or 37 or 38 or 39 or 40 or 41 or 42 or 43 or 44 or 45 or 46 or 47 or 48 or 49 or 50 or 51 or 52 or 53 or 54 or 55 (9566082)
57 clinical status.mp. (28461)
58 "length of stay"/ (299383)
59 bed days.mp. (4872)
60 side effect/ (403613)
61 "quality of life"/ (689264)
62 daily life activity/ or activities of daily life.mp. (125766)
63 activities of daily living.mp. (55129)
64 long term effects.mp. (52781)
65 late effects.mp. (11515)
66 exercise recovery/ or recovery.mp. (809062)
67 functional recovery.mp. (39338)
68 functional assessment/ or functional mobility.mp. (77354)
69 motor performance/ (97958)
70 nutritional status/ (88286)
71 body weight gain/ or body weight change/ or body weight loss/ or body weight variation/ or body weight/ or body weight management/ or body weight maintenance/ or body weight control/ or molecular weight/ or weight/ or muscle weight/ or body weight fluctuation/ (794847)
72 body composition/ (84812)
73 body size/ (32023)
74 body mass/ (704180)
75 height/ or body height/ (142964)
76 growth/ or linear growth.mp. (40939)
77 body composition/ (84812)
78 muscle mass/ (43008)
79 fat free mass/ (8290)
80 fat mass/ (26728)
81 deconditioning/ (820)
82 endurance/ (29154)
83 cardiorespiratory fitness/ (10342)
84 cardiac function.mp. or heart function/ (115236)
85 cardiac ejection.mp. (968)
86 fraction.mp. (681309)
87 fractional.mp. (114389)
88 fractional shortening/ (3262)
89 strength/ (11272)
90 prevention/ (317971)
91 preventative.mp. (32268)
92 graft preservation/ or preservation/ (22441)
93 57 or 58 or 59 or 60 or 61 or 62 or 63 or 64 or 65 or 66 or 67 or 68 or 69 or 70 or 71 or 72 or 73 or 74 or 75 or 76 or 77 or 78 or 79 or 80 or 81 or 82 or 83 or 84 or 85 or 86 or 87 or 88 or 89 or 90 or 91 or 92 (4951593)
94 14 and 56 and 93 (26798)
95 limit 94 to (infant or child or preschool child or school child or adolescent) (4712)
96 from 95 keep 8,195,204,237,278,313,339 (7)
97 from 95 keep 1465,2606 (2)
98 from 95 keep 2987,3400 (2)
99 96 or 97 or 98 (11)
100 14 and 15 (39)
101 99 or 100 (50)
102 from 101 keep 2-10,17,21-22,24-26,28-29,31,33-36,38,40-42,44,46 (28)
